# Tiny giants in a big ocean

**DOI:** 10.1128/spectrum.02992-25

**Published:** 2026-01-06

**Authors:** Victoria F. Queiroz, Abdeali M. Jivaji, Frank O. Aylward

**Affiliations:** 1 Department of Biological Sciences and Center for Emerging, Zoonotic, and Arthropod-borne Pathogens, Virginia Tech, Blacksburg, Virginia, USA; University of Mississippi, University, Mississippi, USA

**Keywords:** *Nucleocytoviricota*, prasinoviruses, algal viruses, microbial oceanography

## Abstract

Prasinoviruses infect some of the most abundant photosynthetic eukaryotes in the ocean, shaping microbial dynamics and playing a major role in nutrient cycling. Despite their ecological significance, they have only been explored in a few marine systems. In a recent study, A. B. de Silva, S. W. Polson, C. R. Schvarcz, G. F. Steward, and K. F. Edwards (Microbiol Spectr 13:e02583-24, 2025, https://doi.org/10.1128/spectrum.02583-24) describe four new prasinoviruses isolated from the tropical North Pacific. The comparative analyses highlighted the placement of these viruses within known prasinovirus lineages while also revealing novel genetic features. To assess their ecological reach, the authors mapped metagenomic reads from global data sets, demonstrating that these viruses are not confined to the Pacific but are globally distributed. These findings expand our understanding of prasinovirus diversity and evolution and highlight their widespread occurrence across oceanic regions.

## COMMENTARY

Viruses are the most abundant biological entities on Earth, with an estimated 4 × 10³⁰ particles globally (1). Given that more than 70% of the planet is covered by oceans, it is unsurprising that seawater contains staggering viral loads (2). Within this immense diversity, double-stranded DNA viruses of the phylum *Nucleocytoviricota*, which includes many lineages of “giant viruses,” are among the most abundant in the ocean (3, 4). Among the *Nucleocytoviricota*, prasinoviruses play a particularly central role in surface waters by infecting members of the *Mamiellophyceae*, a ubiquitous class of unicellular green algae that are among the most widespread marine primary producers (5). Marine phytoplankton are fundamental to global biogeochemical cycles, contributing nearly half of Earth’s primary production. Prasinoviruses represent a substantial fraction of the marine eukaryotic virome (6). By regulating their algal hosts, they influence population dynamics, shape community composition, drive nutrient cycling, and affect carbon export to the deep sea, with outcomes depending on host interactions and ecosystem context (7, 8). Despite their ecological significance, prasinoviruses remain relatively understudied when compared to other abundant oceanic viruses, such as cyanophages.

In a recent study, de Silva et al. (9) characterized four newly isolated prasinoviruses, sequenced and annotated their genomes, thereby doubling the number of available genomes for Micromonas dsDNA viruses. Although the four viruses were obtained from tropical North Pacific waters at relatively close sampling sites, each was isolated using a different *Micromonas* strain as host. Interestingly, all the viruses had a broad host range, capable of infecting strains of *Micromonas* other than their host strain of isolation. The isolates McV-KB2 and McV-KB3 lysed six of the seven tested cell strains, whereas McV-KB4 and McV-SA1 infected only two cell strains. Overall, only one cell strain was permissive to all four viruses.

Following genome sequencing and annotation, the four assemblies revealed both conserved and novel features of prasinoviruses. The genomes averaged 209 kbp in length, with a GC content of approximately 41%, except for McV-KB2, which reached nearly 45%. Synteny analysis indicated that McV-KB2 also displayed the greatest degree of genomic reorganization, whereas the other three showed high structural similarity. As expected, the viruses encode a conserved set of core genes involved in replication, structural functions, and viral metabolism. A quarter of the genome was significantly associated with host genus identity, suggesting potential mechanisms of adaptation to specific host ranges and involvement in host-virus coevolution. In addition, each genome contains distinct accessory regions. Notably, McV-KB2 carried the highest number of unique genes (46), followed by McV-KB3 (36), while McV-SA1 and McV-KB4 harbored fewer unique genes and shared a larger proportion of their gene content with each other.

Prasinoviruses can be divided into three groups according to the marine picophytoplankton they infect: those that infect *Bathycoccus*, those that infect *Micromonas*, and those that infect *Ostreococcus*. Viruses infecting *Bathycoccus* and *Ostreococcus* each form distinct monophyletic clades, whereas those infecting *Micromonas* are paraphyletic. In agreement with these observations, phylogenetic analysis placed McV-KB3, McV-KB4, and McV-SA1 within a single clade, with McV-KB4 and McV-SA1 being the most closely related. This clade clusters with a larger group that includes all previously described *Micromonas*- and *Ostreococcus*-infecting viruses. In contrast, McV-KB2 diverges substantially from the other *Micromonas*-infecting prasinoviruses. Its closest relatives are the *Bathycoccus*-infecting viruses, from which it branched shortly after the divergence of the last common ancestor of all prasinoviruses.

Perhaps, the most striking result is that these prasinoviruses show evidence of global distribution. They were isolated from Station ALOHA in the North Pacific subtropical gyre (NPSG), a representative station of the largest contiguous ecosystem on Earth and a key “proving ground” for microbial ecology studies (10). The NPSG plays an important role in Earth’s nutrient cycles, and microbial dynamics examined here are potentially widespread in oligotrophic waters throughout the global ocean. Read mapping analyses revealed that closely related prasinovirus populations occur across multiple ocean basins, consistent with previous work using PCR-based surveys that demonstrated the ubiquity of these viruses in aquatic systems (11–13). Notably, McV-SA1 accounted for more than half of all Hawai’i Micromonas commoda Viruses (HiMcV) hits in the metagenomic data sets and was the only HiMcV detected in both the North Atlantic and North Pacific basins. In contrast, despite being a sister to McV-SA1, McV-KB4 was underrepresented, with only three hits. This suggests that relatively small genomic differences between otherwise closely related viruses can profoundly influence ecological outcomes.

The discovery and characterization of four new prasinoviruses from the tropical North Pacific mark an important step forward in cataloging the genomic and ecological diversity of algal viruses, reinforcing the central role of eukaryotic algal viruses in the activities of marine ecosystems. By expanding the known diversity of prasinoviruses and documenting their widespread distributions, this study illustrates the power of integrating isolation approaches, which enable detailed analyses of viral genomes and host interactions, with environmental metagenomics, which places these discoveries within a broader ecological context. This dual strategy reveals the global significance of locally isolated viruses and bridging bench virology with oceanography. The identification of novel genes opens avenues for functional studies using transcriptomics, proteomics, and metabolomics to unravel how prasinoviruses evolve and shape microbial communities under changing environmental conditions. In particular, the presence of unique metabolic and host-manipulation genes can also shed light into the sophisticated strategies viruses use to reprogram host physiology to maximize replication, with potential consequences for primary production and carbon cycling. Taken together, these findings not only expand our understanding of the ecological roles of marine viruses but also establish a foundation for future research into their evolutionary history, ecosystem impacts, and possible biotechnological applications.

As the known virosphere expands, so too will our ability to link viruses to their roles in global-scale processes. Closer examination of these “tiny giants” in the ocean will be an important step toward this goal.
